# Risk of non-melanoma skin cancer with biological therapy in common inflammatory diseases: a systemic review and meta-analysis

**DOI:** 10.1186/s12935-021-02325-9

**Published:** 2021-11-22

**Authors:** Ruolin Liu, Qianyi Wan, Rui Zhao, Haitao Xiao, Ying Cen, Xuewen Xu

**Affiliations:** 1 Department of Burn and Plastic Surgery, West China Hospital of Sichuan University, No 37 Wainan Guoxue Road, Chengdu, 610041 China; 2 Department of Gastrointestinal Surgery, West China Hospital of Sichuan University, Chengdu, 610041 China

**Keywords:** Non-melanoma skin cancer, Inflammatory bowel disease, Psoriasis, Rheumatoid arthritis, Biologics

## Abstract

**Background:**

Most previous studies compared the risk for non-melanoma skin cancer (NMSC) in biologic-treated common inflammatory diseases with the general population. Whether the increased NMSC risk is caused by the disease itself, the biologics, or both remains unknown.

**Methods:**

We systematically searched PubMed, Embase, Medline, Web of Science, and Cochrane Library from inception to May 2021. Studies were included if they assessed the risk of NMSC for rheumatoid arthritis (RA), inflammatory bowel disease (IBD), or psoriasis patients treated with biologics compared with patients not receiving biologics. Pooled relative risks (RRs) and 95% confidence intervals (CIs) were calculated using the fixed- or random-effects model.

**Results:**

The current meta-analysis included 12 studies. Compared with patients with the inflammatory disease without biologics, patients receiving biological therapy were associated with an increased risk for NMSC (RR 1.25, 95% CI 1.14 to 1.37), especially in patients with RA (RR 1.24, 95% CI 1.13 to 1.36) and psoriasis (RR 1.28, 95% CI 1.07 to 1.52), but not in patients with IBD (RR 1.49, 95% CI 0.46 to 4.91). The risks for squamous cell skin cancer and basal cell skin cancer were both increased for patients receiving biologics. However, the risk of NMSC did not increase in patients treated with biologics less than 2 years.

**Conclusions:**

Current evidence suggests that increased risk of NMSC was identified in RA and psoriasis treated with biologics compared with patients not receiving biologics, but not in patients with IBD. The inner cause for the increased risk of NMSC in IBD patients should be further discussed.

**Supplementary Information:**

The online version contains supplementary material available at 10.1186/s12935-021-02325-9.

## Background

Rheumatoid arthritis (RA), inflammatory bowel disease (IBD), and psoriasis are three common immune-mediated inflammatory diseases involved with epithelial or connective tissue with overlapping genetic susceptibility and relatively high incidence [1–4]. Previous studies found that all these three diseases increased cancer incidence in epithelial or connective tissue, including melanoma and non-melanoma skin cancer (NMSC) [5–10]. It is worth noting that these three diseases also have overlapping treatment modalities, and they are characterized by long-term treatment [4]. Biologics, including tumor necrosis factor inhibitors (TNFIs; adalimumab, etanercept, infliximab), CD20 inhibitor (rituximab), antagonists of the IL-17 pathway (ustekinumab, secukinumab, and ixekizumab), and antagonists of the IL-6 pathway (tocilizumab) were licensed for the treatment of RA, IBD, or psoriasis in the early part of the last decade [11–14]. Although these drugs are widely used and their efficacy is well-proven, their role in the risk of developing a variety of cancers remains unclear [15]. The above results raise a question, namely, whether the diseases increased the risk for NMSC, or the use of biological agents increased this risk, or both.

Esse et al. identified no significant association between the risk of melanoma and biological treatment for patients with common inflammatory diseases compared with those receiving non-biological therapy, which showed that biological therapy is not critical in developing melanoma for patients with inflammatory diseases [16]. Van Lümig et al. found that patients with psoriasis had a 5.5 (95% confidence interval [CI], 2.2 to 13.4) higher rate of NMSC compared with patients with RA with correction for the duration of TNFIs and other systemic therapies [17]. Therefore, the impact of biological therapy on these three diseases for the occurrence of NMSC should be further investigated to determine the inner relationship between the increasing incidence of NMSC and biologically treated inflammatory diseases.

Previous studies in biologic-treated IBD and psoriasis have found an elevated incidence of NMSC in pan-cancer research [18–20], while these studies selected the general population as a comparison, which cannot distinguish whether the increased risk of NMSC comes from diseases or biological agents. At present, a meta-analysis evaluating the risk of NMSC in biologic-treated patients versus non-biologic-treated patients has been confined to RA patients [21, 22]. A systemic review identified that TNFIs therapy is associated with increased risk of NMSC versus non-TNFIs therapy (relative risk [RR], 1.28; 95% CI 1.19 to 1.38) [22]; other kinds of biologics were not included in the analysis [23]. The risk of NMSC in individuals with IBD or psoriasis treated with biological treatment versus those not receiving biological therapy is even less apparent. A systemic review on the occurrence of any cancer associated with the use of TNFIs for IBD therapy included the studies about NMSC compared with the general population, and no effect size calculation was performed [19, 24]. To the best of our knowledge, the only meta-analysis of any malignancy amongst biologic-treated psoriasis patients examined the risk of NMSC also compared with the general population [18].

To address the above issues, we performed a systematic review evaluating the risk of NMSC in patients with common inflammatory diseases treated with biologics. Considering the inherent relevance of the three diseases, connective studies are necessary to clarify whether the source of the increased risk of NMSC is related to biological agents, the diseases, or both. We aimed to present a therapeutically meaningful review of the available information to help clinicians make better therapy decisions.

## Methods

### Data sources and searches

The following terms were used to search PubMed, Embase, Medline, Web of Science, and Cochrane Library from their creation to May 2021 for eligible studies, with no language constraints: “arthritis rheumatoid” or “rheumatoid arthritis” or “rheumatoid chronic arthritis” or “inflammatory bowel disease*” or “ulcerative colitis” or “crohn” or “psoriasis” or “inflammatory disease*” or “immune-mediated disease*,” “skin cancer*” or “skin neoplasm*” or “NMSC,” and “TNFI” or “tumor necrosis factor inhibitor*” or “tumor necrosis factor-α antagonist*” or “TNF-α inhibitor*” or “anti-TNF” or “biologic*” or “infliximab” or “adalimumab” or “etanercept” or “golimumab” or “certolizumab” or “ustekinumab” or “rituximab” or “abatacept” or “tocilizumab” or “natalizumab” or “vedolizumab.” The details of the search strategy are shown in Additional file 3: Supplementary search strategy. We also conducted a hand search from the reference lists of retrieved articles. This systematic review was conducted following the Preferred Reporting Items for Systematic Reviews and Meta-Analyses guidelines (PRISMA) and Meta-analysis of observational studies in Epidemiology guidelines (MOOSE) [23, 25–29]. The protocol for this meta-analysis was registered in the INPLASY database under the number INPLASY202170005.

### Study selection

Randomized clinical trials, cohort studies, and nested case–control studies to investigate the risk of NMSC in patients with RA, IBD or psoriasis were included for further analysis. Studies were deemed potentially eligible if they matched the preset criteria listed below: (1) Studies on people with RA, IBD, or psoriasis; (2) treatment based on biologics; and (3) the risk estimates and 95% CI of NMSC connected with biologics compared with those not receiving biologics. Studies were excluded if they met the following criteria: (1) use of the general population as the comparator; (2) non-clinical studies, such as animal studies; and (3) no relative risk could be extracted. Two researchers independently assessed study eligibility by screening study titles and abstracts and then reading the studies in total. Discrepancies about eligibility were settled by consensus with the third investigator.

### Data extraction and assessment of the methodological quality

Data extraction was performed independently by two reviewers. The following information was extracted from each paper: lead author; publication year; where the study was conducted; data source; study design; types of biological therapy; comparator therapy; treatment duration; disease severity indicators; sample size; effect size data and associated 95% CI; and adjustment variables. The Newcastle–Ottawa Quality Assessment Scale (NOS) was used to evaluate study selection, matching, and outcome of the included studies [30–32].

### Statistical analysis

The relative risk reported in included articles were chosen for inclusion in the meta-analysis. We calculated summary RRs and associated 95% CIs for all outcomes using random- or fixed-effects models [33]. In studies that provided multiple RR estimates, the ones that were corrected for the highest number of confounders were used. We used the Q test to assess heterogeneity in outcomes across studies, and I^2^ statistic was used to quantify it. An I^2^ score of 50% or higher was considered to show significant heterogeneity. In anticipation of clinical heterogeneity, the random-effects model was performed. Begg’s and Egger’s tests were used to investigate publication bias. Subgroup analyses were conducted based on types of NMSC, types of biological therapy, study quality, treatment years, and sample size. All statistical analyses were conducted using Stata statistical software, version 15.1 (StataCorp., College Station, TX, USA).

## Results

### Search results

**Fig. 1:**
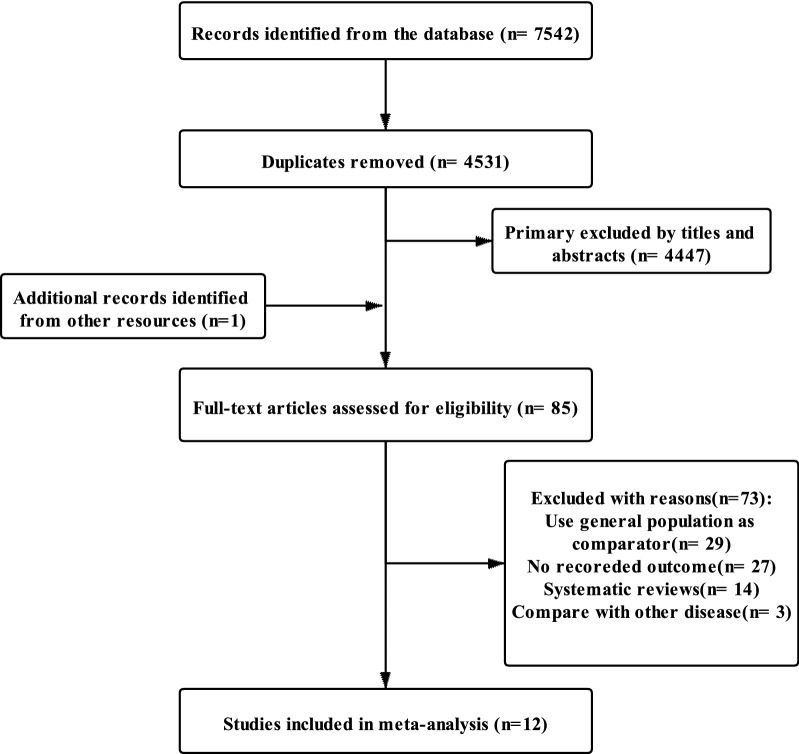
Flow chart for the search and selection of eligible studies

The process of article selection is shown in Fig. 1. The search strategy identified 7542 records from the databases. After removing 3011 duplicates, 4531 records were identified. We excluded 4447 entries by title and abstract screening. The remaining 84 articles and one more paper discovered by hand-searching were read in their entirety and assessed for eligibility. Of these studies, three studies compared different diseases as control, 14 studies were systematic reviews, 27 studies did not report the outcome, and 29 studies used the general population as comparator group. Ultimately, we included 12 articles for analysis.

### Characteristics of included studies

The 12 included studies were published from 2007 to 2019, and all were observational studies conducted in the USA (n = 8), Sweden (n = 2), the UK (n = 1), and Demark (n = 1; Table 1). Amongst these included studies, eight investigations were carried out on patients with RA [9, 34–40], one on patients with IBD [41], two on patients with psoriasis [42, 43], and one on patients with all these three diseases [44]. In all, 109,578 patients were treated with biologics, and 191,062 biologic-naïve patients did not receive biologics. The average duration of treatment varied from 0.3 years to 5.9 years, with research periods spanning 1995 to 2015 (Table 1). The majority of the included studies (n = 11) involved individuals treated with TNFIs [9, 34–39, 41–44]. Amongst them, eight articles only involved TNFIs treatment [9, 34, 36, 37, 39, 41, 43, 44], and three articles reported the independent outcome of adalimumab, etanercept, and infliximab treatment [34, 39, 43]. In addition to TNFIs, individuals treated with rituximab (CD-20 inhibitor), abatacept (CD-28 inhibitor), and tocilizumab (IL-6 inhibitor) were also included in the research [35, 38, 40]. Age and gender adjustments were conducted in all the included studies. Furthermore, adjustment for prior or concurrent immunosuppressive therapy exposure was undertaken in one research [40], and adjustment for race/ethnicity (an indication of skin color, a key risk factor for NMSC) was carried out in four studies [9, 35, 42, 44]. However, UVR exposure was not adjusted in any of the included studies (Table 1).

**Table 1:** General characteristics of the studies

Study	Country	Data source	Study period	Biologic treatment group					None-biologic treatment group					Risk estimate (95%CI)	Adjustment
				Biologic drugs	Number/ cases	Age (years)	Female (%)	Mean/median treatment duration (years)	Control	Number/cases	Age (years)	Female (%)	Mean/median treatment duration (years)		
*RA*															
Dreyer 2013	Denmark	The national Danish DANBIO database	2000–2008	TNFI	3347/42	54.3	73	2.9	Nonbiologic DMARDS	3812/34	61.2	74	2.1	HR,1.10 (0.69–1.76)	Age,gender,calendar time
Wadström 2017	Sweden	The Swedish Rheumatology Quality of Care Register	2006–2015	TNFI	10,760/54	58	74	NR	Conventional systemic DMARDS	46,416/467	64	71	NR	SCC HR,1.09 (0.84–1.42)	Age, gender, start of treatment year, comorbidities, No. of hospitalizations, educational level, days spent in inpatient care
				Abatacept	2016/17	61	80							SCC HR,2.15 (1.31–3.52)	
				Rituximab	3566/24	63	76							SCC HR,1.01 (0.66–1.55)	
				Tocilizumab	1788/5	59	78							SCC HR,0.93 (0.39–2.21)	
Wolfe 2007	USA	US National Data Bank for Rheumatic Diseases	1998–2005	Infliximab	4430/161	58.5	78	2.9	Biologic naïve	NR	58.5	78	NR	OR,1.7 (1.3–2.2)	Age, gender, educational level, smoking history, baseline patient activity scale, baseline prednisone use
				Etanercept	3163/126			2.7						OR,1.2 (1.0–1.5)	
				Adalimumab	812/10			1.2						OR,0.9 (0.5–1.8)	
Ozen 2019	USA	US National Data Bank for Rheumatic Diseases	2005–2015	Abatacept	1099/37	61.5	85.5	2.5	Conventional systemic DMARDS	1103/20	63.3	80.9	2	HR,1.05 (0.22- 4.98)	Age, gender, employment status, annual income, education level, smoking status, disease duration, HAQ-Disability Index, pain and patient global scores, BMI, Rheumatic Diseases Comorbidity Index score, any chronic lung disease, diabetes, number of prior bDMARDs, glucocorticoid use, year of study entry, follow-up time
Solomon 2014	USA	Corrona RA registry	2001–2010	TNFI	3761/22	NR	77.7	NR	Methotrexate	1566/17	NR	72.3	NR	HR,0.57 (0.26–1.27)	Age, gender, race, tobacco, alcohol use, BMI, disease duration, serologic status, joint erosions, CDAI, family history of cancer, HAQ, number of past DMARDs used, use of oral glucocorticoids
				Abatacept	408/5	NR	84.1							HR,1.85 (0.33–10.43)	
				Rituximab	167/1	NR	76.7							HR,1.61 (0.09–28.6)	
Amari 2011	USA	The Austin Information Technology Center and the pharmacy benefits management	1998–2008	TNFI	4088/283	59.6	9.6	NR	Nonbiologic DMARDS	18,396/1043	63.1	9.4	NR	HR,1.42 (1.24–1.63)	Age, gender, race, comorbid diagnoses
Raaschou 2016	Sweden	The Swedish Biologics Register	1998–2012	BCC TNFI	8827/236	55.3	74.8	4.2	BCC Biologic naïve	43,675/1587	61.6	71.5	4.7	HR,1.14 (0.98–1.33)	Age, gender, birth year, country of birth, county of residency, educational level, comorbidities until start of follow-up (hospital admissions/outpatient visits for chronic obstructive pulmonary disease, ischemic heart disease, diabetes mellitus, knee/hip joint replacement surgery, psoriatic disease)
				SCC TNFI	12,558/191	55.2	75.4	5.9	SCC Biologic naïve	46,409/847	60.9	71.5	5.1	HR,1.30 (1.10–1.55)	
Mercer 2017	UK	British Society for Rheumatology Biologics Register	2003–2008	TNFI	11,704/BCC 150, SCC 23	56	76	4.01	Nonbiologic DMARDS	3523/BCC 38, SCC 4	60	72	2.65	BCC HR,1.20 (0.83 to 1.73) SCC HR,1.79 (0.59 to 5.41)	Age,gender
				Etanercept	5086/BCC 57	56	77	3.7						BCC,HR,1.07 (0.70 to 1.63)	
				Infliximab	3663/BCC 59	56	76	2.7						BCC,HR,1.73 (1.14 to 2.62)	
				Adalimumab	5035/BCC 34	57	76	2.05						BCC,HR,0.89 (0.56 to 1.42)	
Haynes 2012	USA	Safety Assessment of Biological Therapeutics	1998–2007	TNFI	19,750/134	NR	85.1	0.5	Methotrexate	9805/77	NR	85.8	0.3	HR,1.07 (0.79–1.46)	Propensity scores calculated according to age, sex, race, residence, nursing home/community dwelling, area income, calendar year, number of hospitalizations, outpatient and emergency room visits, number of different medication classes filled, extraarticular disease manifestations, number of intraarticular and orthopedic procedures, number of laboratory tests ordered for inflammatory markers, chronic obstructive pulmonary disease, diabetes, and use of cancer screening tests
*IBD*															
Haynes 2012	USA	Safety Assessment of Biological Therapeutics	1998–2007	TNFI	2657/14	NR	66.9	NR	Azathioprine or mercaptopurine	3700/30	NR	65.9	NR	HR,0.37 (0.13–1.07)	Propensity scores calculated according to age, sex, race, residence, nursing home/community dwelling, area income, calendar year, number of hospitalizations, outpatient and emergency room visits, number of different medication classes filled, extraarticular disease manifestations, number of intraarticular and orthopedic procedures, number of laboratory tests ordered for inflammatory markers, chronic obstructive pulmonary disease, diabetes, and use of cancer screening tests
Long 2010	USA	PharMetrics Patient- Centric Database	1996–2005	Adalimumab or Infliximab-recent use	387/14	49.7	47.6	NR	None	1548/36	49.2	47.6	NR	CD,HR,2.47 (1.29–4.73)	Age, gender, geographic region, and duration of follow-up
				Adalimumab or Infliximab-persist use	228/7					913/13				CD,HR,3.23 (1.24–8.45)	
*Psoriasis*															
Asgari 2017	USA	Kaiser Permanente Northern California health insurance database	1998–2011	Biologics (including adalimumab, etanercept, infliximab, certolizumab, ustekinumab, golimumab, tocilizumab, abatacept, anakinra, and rituximab)	2285/109	47.6	47	5.86	Nonbiologic therapy	3604/251	53.1	51	5.23	NMSC HR, 1.42 (1.12–1.80) BCC HR, 1.81 (1.23–2.67) SCC HR,1.23 (0.91–1.66)	Age, gender, race/ethnicity, presence of psoriatic arthritis; prior UV light therapy, BMI, and cigarette use
Kimball 2015	USA	MarketScan commercial and Medicare Supplemental claims database	1995–2011	Etanercept	6856/316	49	45.4	NR	Nonbiologic therapy	5857/353	53	55.9	NR	RR, 1.1 (0.8–1.6)	Age, gender, systemic medication exposure at baseline
				Adalimumab	3314/175	49	47.4							RR, 1.2 (0.7–1.9)	
				Infliximab	1044/51	49	53.5							RR, 1.1 (0.4–2.5)	
Haynes 2012	USA	Safety Assessment of Biological Therapeutics	1998–2007	TNFI	563/ < 5	NR	65.2	NR	Methotrexate	735/6	NR	59.6	NR	HR,0.35 (0.04–3.43)	Propensity scores calculated according to age, sex, race, residence, nursing home/community dwelling, area income, calendar year, number of hospitalizations, outpatient and emergency room visits, number of different medication classes filled, extraarticular disease manifestations, number of intraarticular and orthopedic procedures, number of laboratory tests ordered for inflammatory markers, chronic obstructive pulmonary disease, diabetes, and use of cancer screening tests

DMARDs: disease-modifying anti-rheumatic drugs; mHAQ: modified Health Assessment Questionnaire; COPD: chronic obstructive pulmonary disease; BMI: body mass index; CDAI: Clinical Disease Activity Index; CRP: C-reactive protein; DAS28: disease activity score in 28 joints; RDCI: Rheumatic Disease Comorbidity Index score; NR: Not reported

### Quality assessment

According to the NOS, three studies scored 7 and nine studies scored 8, meaning that all the included studies were assessed as high quality[30] (Additional file 1: Table S1). In the selection domain, all the included studies received the highest possible scores (4 of 4). In the comparability domain, all studies scored 1out of 2 for the lack of adjustment for UVR exposure. There was no report on the number of people lost to follow-up or related information in studies from Dreyer, Wadström, and Kimball et al. [36, 38, 43].

### Risk of NMSC

**Fig. 2:**
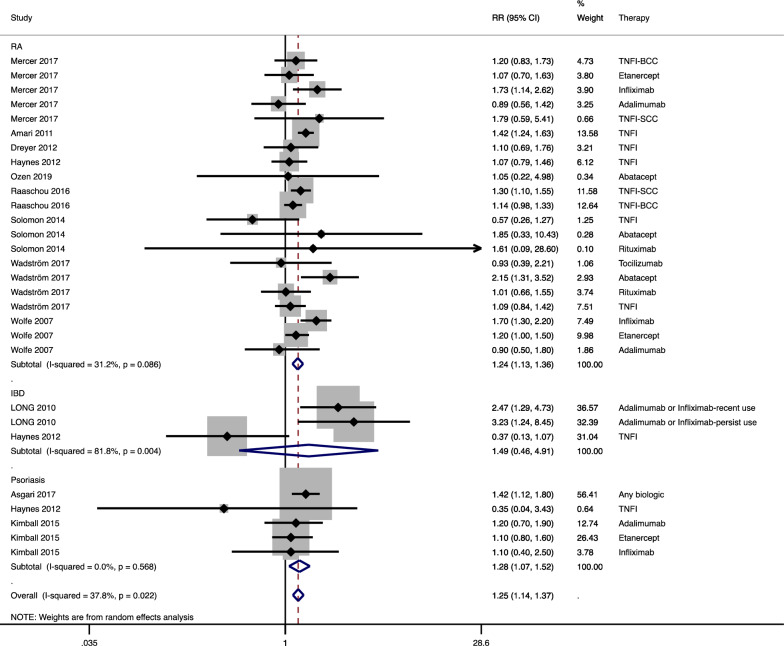
Forest plot for the risk of non-melanoma skin cancer (NMSC) in rheumatoid arthritis (RA), inflammatory bowel disease (IBD), and psoriasis patients receiving biologics compared with patients not receiving biologics. TNFI: tumor necrosis factor inhibitor; BCC: basal cell skin cancer; SCC: squamous cell skin cancer; RR: relative risk; CI: confidence interval. The shadow boxes represent point estimates, and the horizontal lines represent 95% CIs. The weight of the research is reflected by the size of the box. Diamonds represent pooled estimates, with their tips representing 95% CIs

**Fig. 3:**
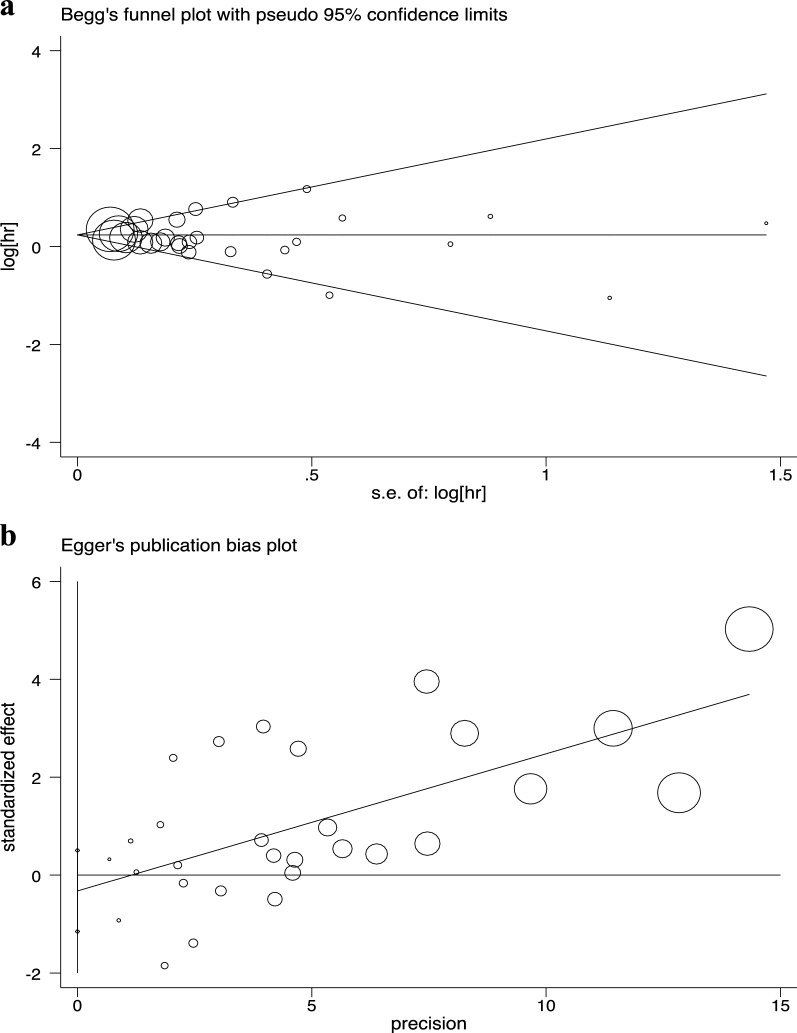
Publication bias. **a** Begg’s test (*P* = 0.99); **b** Egger’s test (*P* = 0.43)

A random-effects model was used to calculate the summary RR of NMSC for patients treated with biologics versus those that did not receive biological drugs. The meta-analysis revealed that the use of biologics was linked with a greater risk of NMSC compared with no use of biologics in three common inflammatory diseases (RR 1.25, 95% CI 1.14 to 1.37; Fig. 2). Amongst them, biological therapy also increased the risk of NMSC in patients with RA (RR 1.24, 95% CI 1.13 to 1.36) and psoriasis (RR 1.28, 95% CI 1.07 to 1.52), but not the risk of those with IBD (RR 1.49, 95% CI 0.46 to 4.91). Heterogeneity was not significant in the RA (I^2^ = 31.2%) and psoriasis (I^2^ = 0.0%) subgroups. No publication bias was indicated in the included studies (Begg *P* = 0.99; Egger *P* = 0.43; Fig. 3).

### Subgroup analysis

To analyze the consistency of results for connections between the usage of biologics and the risk of NMSC, as well as identifying potential causes of inter-study heterogeneity, we conducted subgroup analysis based on the types of NMSC, types of biological therapy, study quality, treatment years, and sample size in the models (Table 2). In the subgroup analysis by type of NMSC, biologics both raised the risk for patients with basal cell skin cancer (BCC; RR 1.16, 95% CI 1.02 to 1.32) and squamous cell skin cancer (SCC; RR 1.34, 95% CI 1.10 to 1.63; Additional file 2: Fig.S1a). However, subgroup analysis based on types of biologics demonstrated that both TNFIs (RR 1.23, 95% CI 1.10 to 1.37) and abatacept (RR 2.00, 95% CI 1.27 to 3.15) increased the risk of NMSC in patients with related inflammatory diseases, while rituximab (RR 1.02, 95% CI 0.67 to 1.56) did not show the same trend (Additional file 2: Fig. S1b). Furthermore, treatment with biologics for more than 2 years increased the risk of NMSC compared with non-biological treatments (RR 1.26, 95% CI 1.17 to 1.37), whereas no significant association was found for receiving biologics less than 2 years and the risk of NMSC (RR 1.04, 95% CI 0.79 to 1.37; Additional file 2: Fig. S1c). Also, regardless of study quality or sample size, patients who used biologics had a higher incidence of NMSC than those who did not use biologics (Additional file 2: Fig. S1d and S1e).

**Table 2:** Association between biological therapy and risk of NMSC in subgroup meta-analyses

Subgroup	No. of studies	RR(95% CI)	I^2^ value (%)	*P* value
All studies	12	1.25 (1.14,1.37)	37.8	0.022
Type of NMSC				
Squamous cell skin cancer	4	1.34 (1.10,1.63)	44.2	0.096
Basal cell skin cancer	3	1.16 (1.02,1.32)	0.0	0.893
Type of biologic therapy				
TNFI	10	1.23 (1.10,1.37)	45.2	0.017
Abatacept	3	2.00 (1.27,3.15)	0.0	0.689
Rituximab	2	1.02 (0.67,1.56)	0.0	0.754
Treatment years				
Less than 2 years	2	1.04 (0.79,1.37)	0.0	0.633
More than 2 years	6	1.26 (1.17,1.37)	19.9	0.248
Study quality				
NOS score = 7	3	1.16 (1.00,1.35)	0.4	0.426
NOS score = 8	9	1.29 (1.15,1.44)	42.8	0.028
Sample size				
< 10,000	12	1.26 (1.12,1.42)	45.0	0.009
≥ 10,000	4	1.21 (1.07,1.36)	0.0	0.667

NMSC: non-melanoma skin cancer; TNFIs: tumor necrosis factor inhibitors; NOS: Newcastle–Ottawa Quality Assessment Scale; RR: relative risk; CI: confidence interval

## Discussion

In the current meta-analysis, a significant association was identified between biological drug exposure and the development of NMSC in patients with three immune-mediated inflammatory diseases. Further analysis demonstrated that biologic-treated patients with RA and psoriasis, but not patients with IBD, had a higher incidence of NMSC compared with patients treated with non-biological therapy. Based on previous studies, the most important alternate options for these patients could be their non-biological comparators, including methotrexate (MTX), hydroxychloroquine, sulfasalazine, or leflunomide for RA patients and MTX, cyclosporine, ciclosporin, hydroxyurea, mycophenolate mofetil, sulfasalazine, or thioguanine for psoriasis patients [42, 44]. These drugs should be considered for patients prone to NMSC, such as patients with red hair color or a family history of NMSC [45, 46]. Recently, evidence has emerged that non-biological therapies are not inferior to biological treatments for RA patients, especially when administered early in the disease’s course [47–50]. Furthermore, considering the high costs and risk of serious infections [51, 52], the use of biologics should be cautious.

To the best of our knowledge, this work is the first meta-analysis to explicitly investigate the risk of NMSC in patients with IBD and psoriasis who received biological therapy compared with their biologic-naïve patients. A meta-analysis of any cancer reported an increased risk of NMSC in patients with IBD without assessing the effect of any treatments [53]. At present, only one other pan-cancer systematic review involved the relationship between biologics-treated IBD patients and the risk of NMSC [19]. However, the comparator group included in this study was the general population and no meta-analysis was performed. The lack of a biologic-naïve comparison group for patients with IBD in two studies left unresolved problems of whether the observed results are due to the disease, the treatments, or both [54]. As we only included the studies that directly compared biologic-treated IBD patients with biologic-naive IBD patients, our study offers a more rigorous and clinically relevant estimate of the risk for NMSC in biologic-treated IBD patients. In this study, no significant association was found between IBD patients treated with biologics and the increased risk of NMSC; therefore, other key factors related to the increased risk of NMSC in IBD patients should be discussed, such as thiopurines treatment [55]. Considering the significant variation of the results between the only two included studies, more relevant original studies are needed in future studies to further clarify this issue [41, 44].

At present, the only systemic review of the pan-cancer study involving the risk of NMSC in patients with psoriasis also treated the general population as comparator group without estimating relevant effect, which demonstrated an increased risk of NMSC in patients with biologic-treated psoriasis compared with the general population [18]. In the current study, we included three studies that reported the NMSC risk on patients with biologic-treated psoriasis compared with the non-biologic-treated patients, which showed an increased risk of NMSC in the meta-analysis.

Previous meta-analyses have indicated a relationship between the risk of NMSC and biological treatment in RA patients, which yielded similar results to our study. Xie et al. summarized four studies and demonstrated that patients with biologic-treated RA had a higher risk of NMSC (RR 1.26, 95% CI 1.09 to 1.45) compared with non-biologic treated individuals [21]. The data by Wang et al. indicated that TNFIs therapy in patients with RA is associated with increased risk of NMSC, but only associated with SCC and not associated with BCC [22]. In the current study, we indicated that biological therapy was also significantly related to the development of NMSC both in SCC and BCC.

BCC (about 70%) and SCC (about 25%) are the most prevalent kinds of NMSC [56]. Only two studies separately analyzing BCC and SCC were included in the previous meta-analysis, in which study population was limited to RA patients, and the study was limited to the association between TNFIs treatment and NMSC risk [22]. The current study extended the previous results by including more original studies assessing the risk of BCC and SCC separately. Thus, a more comprehensive analysis was conducted between the impact of biological therapy for three inflammatory diseases and the incidence of the two main subtypes of NMSC.

In subgroup analysis, biological therapy for more than 2 years was significantly associated with the increased risk of NMSC in three inflammatory diseases, while no significant association was found for less than 2 years’ treatment and the increased risk of NMSC, indicating that the risk of NMSC was related to the duration of biological therapy. However, not all the included studies contained the information of mean treatment duration. For IBD patients, the data of mean treatment duration were missed, and 3 months was used as the minimal treatment duration [41], which suggested that numerous patients were treated with biologics for less than 2 years. For patients with biologic-treated RA and psoriasis, the longest mean treatment duration was up to 5.9 years and 5.86 years, respectively. Based on the information collected from the included studies, we infer that treatment duration might partially explain the difference in NMSC risk between the IBD patients’ group and the other two groups.

To further clarify the impact of different biological treatments on the occurrence of NMSC, we conducted a subgroup analysis by different biological treatments. The results showed that TNFIs and abatacept were both associated with the increased risk of NMSC in inflammatory diseases. However, no significant difference was identified between the use of rituximab and the risk of NMSC in RA patients.

TNFIs were the most often used biologics in all the three common inflammatory diseases groups, as shown in Table 1. There are some biological reasons for the relationship between TNFIs therapy and the increased risk of NMSC. TNF is a key cytokine that modulates the inflammatory response and may play a role in tumor formation, which can regulate cell survival, proliferation, and cell death, as well as the transcription of proinflammatory cytokines by activating pathways [57]. TNF can either induce tumor cell death or survival depending on the conditions. The risk of developing NMSC from being treated with TNFIs has been widely studied in patients with RA. A previous meta-analysis based on six original articles also reported an increased risk of NMSC in patients with TNFI-treated RA [22]. In the subgroup analysis of TNFIs treatment, this meta-analysis included more relevant original studies, which increased the robustness and reliability of the results.

As a cytotoxic T-lymphocyte-associated protein 4-fusion protein, abatacept specifically inhibits T cell activation, which has been licensed to treat RA. Abatacept is likely related to an increased risk of cancer as it reduces anti-tumor response and immune surveillance [58, 59]. In the included studies, only three studies involved abatacept, and all were based on RA populations [35, 38, 40]. One in three studies based on 2016 RA patients found a significant association between the abatacept therapy and the risk of NMSC (RR 2.15, 95% CI 1.31 to 3.52) [38]. Although the other two studies did not find a significant relationship between the use of abatacept and the risk of NMSC, their sample sizes were relatively small [35, 40]. Rituximab has been extensively used in lymphoma patients and is now licensed for RA based on growing evidence of effectiveness and short-term safety [60]. However, little is known about the effects of rituximab exposure on the risk of NMSC. The study population of the two included studies involving rituximab only involved patients with RA, including the study published by Wadström et al. [38] in 2017 and the study published by Solomon et al. [35] in 2014. Neither study found a significant relationship between rituximab therapy and the occurrence of NMSC. A previous study found no significant difference in the incidence of NMSC in kidney transplant recipients receiving rituximab treatment compared with the recipients not receiving rituximab treatment, which was consistent with the results of our study [61]. Based on the above results, more and larger studies are needed to analyze the longer safety of biological drugs, especially for abatacept and rituximab.

The severity of the disease could also be an important factor in analyzing the risk of NMSC [62]. Previous studies identified that the severity of RA is related to the risk of non-Hodgkin lymphoma, and the authors explain their findings in terms of increased cumulative inflammatory activity [63]. Moreover, the severity of IBD is associated with the development of colorectal cancer, which is considerably related to the extent of colitis [64, 65]. However, to the best of our knowledge, the severity of the disease has not been considered as the risk factor for the development of NMSC in biologic-treated patients with common inflammatory diseases compared with the patients receiving non-biological treatments. Therefore, the subgroup analysis classified by the severity of the disease could not be conducted. Considering the effect of inflammatory disease severity in other types of cancer, the risk of NMSC might also be influenced. Thus, the severity of the common inflammatory disease should be considered while studying the risk of NMSC in biologic-treated patients in future research.

The following are the strengths of this meta-analysis. First, to decrease the possibility of missing reports, we thoroughly searched five major databases without publication dates or language constrains. Secondly, our analysis followed a predefined protocol to include studies that met rigorous inclusion and exclusion criteria. Thirdly, several stratified analyses were carried out based on several influential study variables, including types of NMSC, types of biological therapy, study quality, treatment years, and sample size. Fourth, all articles included in this study received a relatively high score according to the NOS.

Nevertheless, this meta-analysis had several limitations. The small number of IBD- and psoriasis-specific studies comparing the risk of NMSC between biologic-treated non-biologic treated patients was the major limitation in this meta-analysis. Despite our thorough search, we only found two articles on IBD and three articles on psoriasis qualified for inclusion. Furthermore, the I^2^ value identified significant inter-study heterogeneity in the meta-analysis for IBD patients, which was understandable given the wide range of differences across studies regarding recruited participants, treatment drugs, and other study characteristics. Therefore, we conducted subgroup analysis for different research characteristics, partially explaining the heterogeneity among the studies. Also, more studies assessing the association of biological therapy and the incidence of NMSC in patients with IBD and psoriasis are needed.

Moreover, cohort studies have a more significant chance of irreversible bias, mainly confounding, than other types of work. Although all studies corrected for age and gender for NMSC risk, the possibility of bias from unmeasured confounders, which might result in overestimation or underestimating for effect estimate, also remained. For example, the absence of correction for known risk variables for NMSC, including UVR exposure and race/ethnicity, was observed in the studies included in this analysis.

Additionally, although no conclusive evidence of publication bias was found based on the Begg’s and Egger’s tests, as we did not search for unpublished articles or other such literature, we cannot entirely rule out the potential of publication bias. Finally, the estimates were based on 12 studies from Europe or the US, while data from other regions, such as Asia and Africa, were inaccessible. Therefore, we need to be cautious in generalizing the findings in this meta-analysis to other regions’ populations.

## Conclusion

This study found a positive association between biological therapy and the increased development of NMSC in patients with RA and psoriasis but not in patients with IBD compared with patients not receiving biological therapy. Therefore, biological therapy might be avoided in patients with RA or psoriasis who are at high risk of NMSC. The inner cause for the increased risk of NMSC in IBD patients should be further discussed. Considering the significant heterogeneity of IBD in previously published studies, we propose that further large, well-designed studies on this issue are warranted to enhance assurance. The main risk factors for NMSC should also be taken into consideration in future studies.

## Supplementary Information

**Additional file 1: Table S1.** Methodological quality of studies included in the final analysis based on the Newcastle–Ottawa Scale.

**Additional file 2: Fig. S1.** Forest plot for the subgroup analysis of the risk for non-melanoma skin cancer (NMSC) in patients with common inflammatory diseases receiving biologics compared with patients not receiving biologics classified by (a) types of NMSC, (b) types of biological treatments, (c) treatment years, (d) Newcastle–Ottawa Quality Assessment Scale (NOS) score, and (e) sample size. RA: rheumatoid arthritis; IBD: inflammatory bowel disease; TNFI: tumor necrosis factor inhibitor; BCC: basal cell skin cancer; SCC: squamous cell skin cancer; RR, relative risk; CI, confidence interval. The shadow boxes represent point estimates, and the horizontal lines represent 95% CIs. The weight of the research is reflected by the size of the box. Diamonds represent pooled estimates, with their tips representing 95% CIs

**Additional file 3.** Detailed search strategy.

## Data Availability

All data generated or analyzed during this study are included in this article [and its supplementary information files].

## Abbreviations

BCC: Basal cell skin cancer
BMI: Body mass index
CDAI: Clinical Disease Activity Index
CI: Confidence interval
DMARDs: Disease-modifying anti-rheumatic drugs
HAQ: Health Assessment Questionnaire
IBD: Inflammatory bowel disease
MOOSE: Meta-analysis of observational studies in Epidemiology guidelines
MTX: Methotrexate
NMSC: Non-melanoma skin cancer
NOS: Newcastle–Ottawa Quality Assessment Scale
PRISMA: Preferred Reporting Items for Systematic Reviews and Meta-Analyses guidelines
RA: Rheumatoid arthritis
RR: Relative risk
SCC: Squamous cell skin cancer
TNFI: Tumor necrosis factor inhibitor
