# Supplementary material for: Risk of non-melanoma skin cancer with biological therapy in common inflammatory diseases: a systemic review and meta-analysis
Source: Cancer Cell Int. 2021 Nov 22;21:614. doi: 10.1186/s12935-021-02325-9 (PMC8607648; doi:10.1186/s12935-021-02325-9)
Supplement: Supplementary file 1 — Additional file 1: Table S1. Methodological quality of studies included in the final analysis based on the Newcastle–Ottawa Scale. [file 12935_2021_2325_MOESM1_ESM.docx]

**Supplementary table 1**

**Table S1 Methodological quality of studies included in the final analysis based on the Newcastle-Ottawa Scale**

| **Study** | **Selection (maximum one star per item)** | | | | **Comparability (maximum two stars)** | **Outcome (maximum one star per item)** | | | **Total score** |
| --- | --- | --- | --- | --- | --- | --- | --- | --- | --- |
|  | **Representativeness of exposed cohort** | **Selection of non-exposed cohort** | **Ascertainment of exposure** | **Outcome not present at baseline** | **Comparability** | **Assessment of outcome** | **Length of follow -up** | **Adequacy of follow-up** |  |
| **Mercer 2017** | * | * | * | * | * | * | * | * | 8* |
| **Dreyer 2012** | * | * | * | * | * | * | * | / | 7* |
| **Haynes 2012** | * | * | * | * | * | * | * | * | 8* |
| **Ozen 2019** | * | * | * | * | * | * | * | * | 8* |
| **Raaschou 2016** | * | * | * | * | * | * | * | * | 8* |
| **Solomon 2014** | * | * | * | * | * | * | * | * | 8* |
| **Wadström 2017** | * | * | * | * | * | * | * | / | 7* |
| **Wolfe 2007** | * | * | * | * | * | * | * | * | 8* |
| **Asgari 2017** | * | * | * | * | * | * | * | * | 8* |
| **Kimball 2015** | * | * | * | * | * | * | * | / | 7* |
| **Amari 2011** | * | * | * | * | * | * | * | * | 8* |
| **Long 2010** | * | * | * | * | * | * | * | * | 8* |
