# Supplementary material for: Risk of non-melanoma skin cancer with biological therapy in common inflammatory diseases: a systemic review and meta-analysis
Source: Cancer Cell Int. 2021 Nov 22;21:614. doi: 10.1186/s12935-021-02325-9 (PMC8607648; doi:10.1186/s12935-021-02325-9)
Supplement: Supplementary file 2 — Additional file 2: Fig. S1. Forest plot for the subgroup analysis of the risk for non-melanoma skin cancer (NMSC) in patients with common inflammatory diseases receiving biologics compared with patients not receiving biologics classified by (a) types of NMSC, (b) types of biological treatments, (c) treatment years, (d) Newcastle–Ottawa Quality Assessment Scale (NOS) score, and (e) sample size. RA: rheumatoid arthritis; IBD: inflammatory bowel disease; TNFI: tumor necrosis factor inhibitor; BCC: basal cell skin cancer; SCC: squamous cell skin cancer; RR, relative risk; CI, confidence interval. The shadow boxes represent point estimates, and the horizontal lines represent 95% CIs. The weight of the research is reflected by the size of the box. Diamonds represent pooled estimates, with their tips representing 95% CIs [file 12935_2021_2325_MOESM2_ESM.docx]

**Fig.S1 Forest plot for the subgroup analysis of the risk for non-melanoma skin cancer (NMSC) in patients with common inflammatory diseases receiving biologics compared with patients not receiving biologics classified by** (a) types of NMSC, (b) types of biological treatments, (c) treatment years, (d) Newcastle-Ottawa Quality Assessment Scale (NOS) score, and (e) sample size. RA: rheumatoid arthritis; IBD: inflammatory bowel disease; TNFI: tumor necrosis factor inhibitor; BCC: basal cell skin cancer; SCC: squamous cell skin cancer; RR, relative risk; CI, confidence interval. The shadow boxes represent point estimates, and the horizontal lines represent 95% CIs. The weight of the research is reflected by the size of the box. Diamonds represent pooled estimates, with their tips representing 95% CIs
