# Supplementary material for: Risk of non-melanoma skin cancer with biological therapy in common inflammatory diseases: a systemic review and meta-analysis
Source: Cancer Cell Int. 2021 Nov 22;21:614. doi: 10.1186/s12935-021-02325-9 (PMC8607648; doi:10.1186/s12935-021-02325-9)
Supplement: Supplementary file 3 — Additional file 3. Detailed search strategy. [file 12935_2021_2325_MOESM3_ESM.docx]

**Search strategies**

**Pubmed**

(((((((((((((((((Psoriasis[MeSH Terms]) OR (Psoriasis)) OR (Arthritis, Rheumatoid)) OR (Arthritis, Rheumatoid[MeSH Terms])) OR (rheumatoid arthritis)) OR (rheumatoid chronic arthritis)) OR (Colitis, Ulcerative[MeSH Terms])) OR (Crohn Disease[MeSH Terms])) OR (Inflammatory Bowel Diseases[MeSH Terms])) OR (Colitis, Ulcerative)) OR (Ulcerative colitis)) OR (Crohn)) OR (inflammatory bowel diseases)) OR (inflammatory bowel disease)) OR (immune-mediated disease)) OR (immune-mediated diseases)) AND ((((((Skin Neoplasms[MeSH Terms]) OR (skin cancer)) OR (Skin Neoplasms)) OR (skin cancers)) OR (Skin Neoplasm)) OR (NMSC))) AND (((((((((((((((((((((((((Infliximab[MeSH Terms]) OR (Etanercept[MeSH Terms])) OR (Adalimumab[MeSH Terms])) OR (Certolizumab Pegol[MeSH Terms])) OR (Ustekinumab[MeSH Terms])) OR (Rituximab[MeSH Terms])) OR (Abatacept[MeSH Terms])) OR (Natalizumab[MeSH Terms])) OR (infliximab)) OR (etanercept)) OR (adalimumab)) OR (golimumab)) OR (certolizumab)) OR (rituximab)) OR (abatacept)) OR (anakinra)) OR (tocilizumab)) OR (natalizumab)) OR (vedolizumab)) OR (biologic*)) OR (biological therapy)) OR (TNFI)) OR (tumor necrosis factor inhibitor*)) OR (TNF-α inhibitor*)) OR (anti-TNF))

**Web of Science**

(TOPIC: (Psoriasis) OR TOPIC: (Arthritis, Rheumatoid) OR TOPIC: (rheumatoid arthritis) OR TOPIC: (rheumatoid chronic arthritis) OR TOPIC: (Colitis, Ulcerative) OR TOPIC: (Crohn) OR TOPIC: (Ulcerative colitis) OR TOPIC: (inflammatory bowel diseases) OR TOPIC: (inflammatory bowel disease) OR TOPIC: (immune-mediated disease) OR TOPIC: (immune-mediated diseases) ) AND (TOPIC: (Skin Neoplasms) OR TOPIC: (skin cancer) OR TOPIC: (Skin Neoplasms) OR TOPIC: (skin cancers) OR TOPIC: (Skin Neoplasm) OR TOPIC: (NMSC) )AND(TOPIC: (infliximab) OR TOPIC: (etanercept) OR TOPIC: (adalimumab) OR TOPIC: (golimumab) OR TOPIC: (certolizumab) OR TOPIC: (rituximab) OR TOPIC: (abatacept) OR TOPIC: (anakinra) OR TOPIC: (tocilizumab) OR TOPIC: (natalizumab) OR TOPIC: (vedolizumab) OR TOPIC: (biologic*)OR TOPIC: (biological therapy) OR TOPIC: (TNFI) OR TOPIC: (tumor necrosis factor inhibitor*) OR TOPIC: (TNF-α inhibitor*) OR TOPIC: (anti-TNF) )

**Embase AND Medline**

(exp Psoriasis/ OR exp Arthritis, Rheumatoid/ OR exp Colitis, Ulcerative/ OR exp Crohn Disease/ OR exp inflammatory bowel diseases/ OR (Psoriasis or Rheum* or 'Ulcerative colitis' or 'immune-mediated disease*' or Crohn or 'inflammatory bowel disease*').af.) AND (exp Skin Neoplasms/ OR ('skin cancer*' or 'skin neoplasm*' or NMSC).af.) AND (exp infliximab/ OR exp etanercept/ OR exp adalimumab/ OR exp golimumab/ OR exp certolizumab pegol/ OR exp ustekinumab/ OR exp rituximab/ OR exp abatacept/ OR exp anakinra/ OR exp tocilizumab/ OR exp vedolizumab/ OR (infliximab or etanercept or adalimumab or golimumab or certolizumab or ustekinumab or rituximab or abatacept or anakinra or tocilizumab or natalizumab or vedolizumab or 'biologic*' or 'biological therapy' or TNFI or 'tumor necrosis factor inhibitor*' or 'TNF inhibitor*' or anti-TNF).af.)

**Cochrane Library**

(MeSH descriptor: [Psoriasis] explode all trees OR MeSH descriptor: [Arthritis, Rheumatoid] explode all trees OR MeSH descriptor: [Colitis, Ulcerative] explode all trees OR MeSH descriptor: [Crohn Disease] explode all trees OR MeSH descriptor: [Inflammatory Bowel Diseases] explode all trees) AND (MeSH descriptor: [Skin Neoplasms] explode all trees OR (NMSC):ti,ab,kw) AND (MeSH descriptor: [Infliximab] explode all trees OR MeSH descriptor: [Etanercept] explode all trees OR MeSH descriptor: [Adalimumab] explode all trees OR MeSH descriptor: [Certolizumab Pegol] explode all trees OR MeSH descriptor: [Ustekinumab] explode all trees OR MeSH descriptor: [Rituximab] explode all trees OR MeSH descriptor: [Abatacept] explode all trees OR MeSH descriptor: [Natalizumab] explode all trees OR (golimumab):ti,ab,kw OR (anakinra):ti,ab,kw OR (tocilizumab):ti,ab,kw OR (vedolizumab):ti,ab,kw OR (biological therapy):ti,ab,kw OR (biological therapy):ti,ab,kw OR (tumor necrosis factor inhibitor*):ti,ab,kw OR (TNF-α inhibitor*):ti,ab,kw OR (TNF-α inhibitor*):ti,ab,kw)
